# Isolated Bilateral Congenital Iris Sphincter Agenesis

**DOI:** 10.1155/2011/479092

**Published:** 2011-12-14

**Authors:** Aparna Rao

**Affiliations:** Glaucoma Services, LV Prasad Eye Institute, Patia, Orissa, Bhubaneswar 751024, India

## Abstract

*Purpose*. We herein report a patient with bilateral congenital total iris sphincter agenesis with no other abnormality detected on systemic examination. *Methods*. A 24-year-old laborer presented to us for a routine checkup with complaint of photophobia and inability to work under sunlight. Examination revealed bilateral absence of sphincter and 6.5 mm pupil in both eyes in the undilated state. *Results*. Accommodation was poor in both eyes. Systemic examination was within normal limits. He was prescribed bifocal photochromic glasses for constant wear. *Conclusions*. Congenital sphincter agenesis can occur in an isolated form without systemic abnormalities which can be managed conservatively.

## 1. Introduction

Congenital mydriasis, a rare congenital anomaly, has been described previously with loss of accommodation, ciliary muscle aplasia [1–3], dilator agenesis, as well with cardiovascular and pulmonary abnormalities like patent ductus arteriosus and cystic lung disease [2, 4–6]. We report a case in a young adult with total sphincter agenesis with lack of any systemic abnormality.

## 2. Case Report

A 24-year-male, a daily wage laborer, presented to us for a routine checkup. He had complaints of photophobia and inability to work under the sunlight for more than two continuous hours.

**Figure 1:**
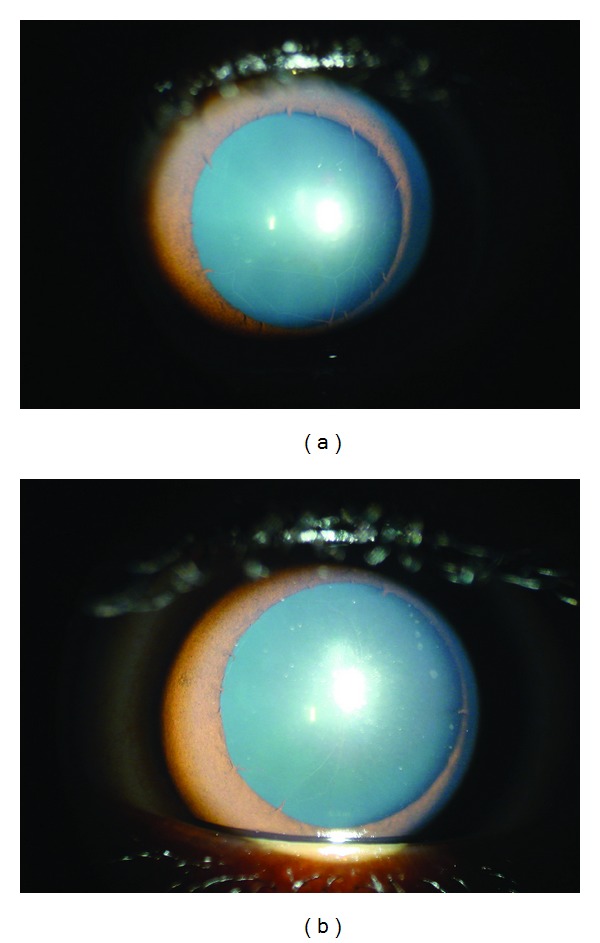
(a) Clinical photograph of the right eye with complete absence of the iris sphincter, persistent pupillary membrane stretching across a large pupil in undilated state. (b) Clinical photograph of the left eye with rudimentary strands arising from the iris margin and a large pupil in undilated state.

On examination, his unaided visual acuity was 20/80, N18 in both eyes. His best corrected visual acuity with glasses improved to 20/20, N12 both eyes (+0.75DC × 180, Right eye, +1DC × 170_0_, with a near add of +2.25DS both eyes). Slit lamp examination showed a 6.5 mm pupil in both eyes. Detailed examination revealed total absence of the iris sphincter and collarette in both eyes (Figures 1(a) and 1(b)). Strands of persistent pupillary membranes were seen extending across the iris margins arising from what appeared to be rudimentary stubs of the collarette (Figure 1(b)). There were no iridolenticular adhesions, and the lens was optically clear in both eyes. His applanation IOP measured 14 mmHg both eyes. Gonioscopy revealed open angles with numerous iris processes.

The pupil in both eyes failed to constrict to an accommodative target or pilocarpine 2%, and reaction to light was very sluggish in both eyes. Phenylephrine instillation caused slight dilatation to 7 mm in both eyes. The patient was unable to fixate on any accommodative target.

Fundus examination revealed normal discs with no other posterior segment pathology.

Review history confirmed the presence of large pupils and same complaints since early childhood. No similar complaints were present in other members of his family. A complete medical examination by a physician and echocardiogram ruled out any systemic anomaly.

 In view of his occupation and poor literary status, he was prescribed photochromic glasses with bifocal correction rather than tinted contact lens. There was obvious increase in the comfort level with reduced photophobia at work with glasses at 1-week review.

## 3. Discussion

Iris sphincter agenesis has been associated with ciliary muscle aplasia with resultant loss of accommodation as well as combined absence of the dilator muscle [1–3]. Our case had loss of accommodation with intact dilators. Conventionally, this condition has been described as part of a syndromic complex involving aneurysmal dilatation of a patent ductus arteriosus or cystic lung disease [1, 4–6]. Family history with the same condition occurring in siblings has also been reported [2, 4]. Our case, however, presented as an isolated ocular anomaly with no systemic or familial associations.

 We believe that our case represented a congenital insult at around 10–14 weeks when the embryogenesis of sphincter muscle commences. This was, therefore, associated with some amount of ciliary muscle aplasia with no other ocular abnormality. We deferred any surgery (pupilloplasty) in this case, keeping in view the young age and the risk of cataract formation postoperatively. Contact lenses were not a viable option either because of the roadside occupation. Therefore, the bifocal correction was preferred which gave him full relief.

## 4. Conclusion

Iris sphincter agenesis is a rare condition that may occur with or without systemic involvement. Isolated involvement may mandate conservative treatment with bifocal glasses or tinted contact lenses.

##  Conflict of Interests

The authors have no financial or proprietary interests in the products used in the study.
